# A prospective cohort study of the relationship of female genital mutilation with birth outcomes in Somalia

**DOI:** 10.1186/s12905-022-01790-2

**Published:** 2022-05-31

**Authors:** Deniz Kulaksiz, Ifrah Abdi Nor, Recep Erin, Kubra Baki Erin, Tuncay Toprak

**Affiliations:** 1 Department of Obstetrics and Gynecology, Kanuni Training and Research Hospital, University of Health Sciences, Trabzon, Turkey; 2 Department of Obstetrics and Gynecology, Recep Tayyip Erdogan Training and Research Hospital, University of Health Sciences, Mogadishu, Somalia; 3 Department of Urology, Fatih Sultan Mehmet Training and Research Hospital, University of Health Sciences, Istanbul, Turkey

**Keywords:** Birth complication, Female genital mutilation, Perineal tears, Somalia

## Abstract

**Background:**

Female genital mutilation (FGM) is defined as the partial or complete removal of the external female genitalia for non-medical reasons. There are some complications related to childbirth that concern both the mother and the baby. In this study, we aimed to evaluate the birth outcomes of FGM, which is widely applied in Somalia.

**Methods:**

The study included 268 women who gave birth at 37–42 weeks of gestation with a cephalic singleton, 134 with FGM and 134 without FGM. This study was designed a prospective cohort study and conducted between January 2019 and December 2020. Patients’ ages, duration of delivery, FGM types, caesarean section requirements, before and after birth hemoglobin levels, birth weeks, baby birth weights and perineal tear data were recorded. In addition, we analyzed neonatal intensive care needs and APGAR scores for infants.

**Results:**

In patients with FGM, it was determined that the outlet obstruction increased 2.33 times, perineal tears increased 2.48 times, the need for caesarean section increased 2.11 times compared to the control group, and the APGAR score below 7 at the 5th minute in the children increased 2 times and the need for neonatal intensive care increased 1.87 times.

**Conclusions:**

FGM causes increased risk of perineal tear, prolongation in the second stage of labour, increased need for emergency caesarean section, and increased need for NICU for infants. Prevention of FGM will help reduce both obstetric and neonatal complications.

## Background

Female genital mutilation (FGM) is defined as the partial or complete removal of the external female genitalia for non-medical reasons such as social pressure, belief in ancestors, being an indicator of virginity before marriage, belief that women’s libido decreased with FGM, and being influenced by other communities in the immediate vicinity [1]. The reasons for its application vary according to regions and time [2]. FGM is practiced because of social pressure, being accepted by the society, being a sign of chastity, preparing their children for marriage, getting rid of the masculine structure of the woman, being recommended by some religious leaders and seen as a cultural tradition [1]. In Sub-Saharan African countries, it used to be mostly practiced by village midwife women, but today it is mostly practiced by health professionals [3]. According to UNICEF, FGM statistics are not known exactly, however, over 200 million women are thought to be affected in at least 30 countries [4].

The classification of FGM according to the World Health Organization (WHO) was given in Table 1 [1].

**Table 1:** FGM classification according to the World Health Organization

Type 1	Partial or total removal of the clitoral glans and/or the clitoral hood
Type 2	Partial or total removal of the clitoral glans and the labia minora with or without removal of the labia majora
Type 3	Narrowing of the vaginal opening through the creation of a covering seal, also called infibulation
Type 4	All other harmful procedures to the female genitalia for non-medical purposes, e.g. incising, piercing, pricking, scraping and cauterizing the genital area

In a 2018 study by WHO covering 27 countries, they reported that the annual cost of FGM complications to be approximately 1.4 billion USD. If the FGM application can be stopped completely, this cost will decrease by 60% within 30 years [1].

Some complications related to FGM, both in the long and short term, have been described. While bleeding, pain, fever, infection and urinary problems are short-term complications, long-term complications include dyspareunia, clitoral cyst, keloid, chronic pelvic infection, anorgasmia, and dystocia [1, 5, 6]. Long-term complications have often been associated with type 3 FGM [7]. After some FGM applications, deinfibulation is required before sexual intercourse or childbirth.

According to 2016 data, the frequency of FGM in Somali women between the ages of 15–49 is reported to be 98%, but there is no data between the ages of 0–15 [4]. Most FGMs are type 2 and type 3 FGM in Somalia[4]. However, there is no information in the literature about the current prevalence and types of FGM in Somalia.

Besides these short- and long-term complications, FGM has complications related to deliver that concern both the mother and the baby. In a review, prolonged hospitalization for the mother, increased frequency of cesarean section, obstructed labour, prolonged 2nd stage of labour and low birth weight for the baby were associated with FGM [8]. In the meta-analysis of 11 articles, the risk ratio for postpartum hemorrhage with FGM, especially in African countries, was reported as 2.59 [1.28, 5.25] [7].

There are many studies on maternal and neonatal complications of FGM. However, these studies are retrospective studies that include a few participants, do not have an appropriate control group, are conducted on immigrant populations or in places where health care providers are of higher quality [7, 9, 10]. Our study is the first study in Somalia to prospectively evaluate the relationship of female genital mutilation with birth complications. In addition, we think it is important that our study is carried out on local people with limited access to health services, rather than immigrants, to reflect the reality.

## Methods

### Study design

This study is a prospective cohort study. The study was conducted at Mogadishu Somalia-Turkey Recep Tayyip Erdogan Training and Research Hospital, which is the only tertiary care hospital in Mogadishu between January 2019 and December 2020. Approval was obtained from the local ethics committee for the study. We protected the privacy of the patients according to the Helsinki Declaration. Patient information was recorded using a coding system so that personal information would not be shared.

### Sample size

G* Power software version 3.01 (Franz Foul, Kiel, Germany) was used for power analysis for two study groups with and without FGM. The female population in Somalia was approximately 7.97 million [11]. For the obstetric consequences in the study of Gebremicheal et al., power analysis software was used by using the mean and number of study participants [12]. While it was 7.1% in the prolonged 2nd stage of labour group without FGM, it ranged from 5.6 to 17.5% in the FGM group. The previous study showed it in the literature that 134 participants in each study arm (total of 268 participants) were required to elicit a medium effect size (d = 0.5) at a significance level of a = 0.05 at 80% power.

### Study population

We included pregnant women who applied to the hospital for delivery in the study. Patients who were pregnant before 37–42 weeks, planned elective cesarean section, had fetal malpresentation, could not get consent for the study, had a body mass index (BMI) above 35 and were in the second stage of labour were not included in the study. Being a mother older than 35 has been associated with complications such as placenta previa, emergency caesarean delivery, postpartum hemorrhage, premature birth, and outlet obstruction [13]. Therefore, we did not include patients with high BMI in the study. We included patients with the 1–1 pairing method to form the FGM group and the control group. In the first stage of labour, 8 patients in the control group and 9 patients in the FGM group were excluded from the study for various reasons, such as cesarean section for maternal request, insufficient financial means of the family. Until the sample size was reached, the women who came for delivery were included in the study by taking their consent.

### Data collection

2 obstetricians and gynecologists and a research assistant collected study data. The patients’ FGM type, age, gravity, prolonged second stage information, outlet obstruction, perineal tear, prenatal hemoglobin (Hb), 8 h postnatal Hb, and need for cesarean section were recorded. We defined the prolonged second stage as lasting longer than 2 h in nulliparas and longer than 1 h in multiparas. Perineal tear is a laceration of the skin and other soft tissue structures at birth and can be spontaneous or iatrogenic, like an episiotomy [14, 15]. We based our study on this definition. Stillbirth, hospitalizations in the neonatal intensive care unit (NICU) of newborns, and appearance, pulse, grimace, activity, respiration (APGAR) scores were also recorded. In the study of Rodriguez et al., having FGM was not defined as an indication for cesarean section [16]. Having FGM was not considered as a cesarean indication in our study.

### Data analysis

All data collection forms were entered SPSS for Windows version 23.0 and analyzed. We analyzed the compatibility of the data with the normal distribution via the Kolmogorov-Smirnov test. We made descriptive statistics for the study variables. The Chi-square test was used for statistical analysis between the FGM group and the control group. We performed bivariate analysis to test the relationship between independent and outcome variables. In the statistical analysis performed at the 95% confidence interval, p < 0.05 was accepted as significant.

## Results

The study included 268 participants, 134 in the control group and 134 in the FGM group. All participants were Somalis and Muslims. Of the patients included in our study, 27 (20%) had type 1 FGM, 81 (61%) had type 2, and 26 (19%) had type 3 FGM.

The maternal age of the participants in our study was 21.4 ± 1.8 years. There was no difference between groups in terms of age, body mass index, birth week, and infant birth weight. Second stage of labour was significantly longer in the FGM group. Although haemoglobin (Hb) levels of patients were similar between groups before delivery, it is significantly lower in the FGM group after delivery. 1st and 5th minute APGAR scores of the babies were significantly lower in the FGM group. We showed the mean values and standard deviation of the data related to both FGM and control groups in the Table 2.

**Table 2:** Maternal and fetal descriptive features and datas

	FGM group (*n* = 134)	Control group (*n* = 134)	*p* value*
Age (years)	21.1 ± 1.7	21.7 ± 1.9	0.453
BMI (kg/m^2^)	26.7 ± 4.1	27 ± 3.9	0.259
Birth week	39.2 ± 1.3	39.2 ± 1.2	0.512
Duration of latent stage (minute)	601.9 ± 186.6	613.3 ± 184.2	0.317
Duration of active stage (minute)	204 ± 87.6	202.9 ± 90.0	0.419
Duration of second stage (minute)	74.3 ± 26.8	45.6 ± 25.4	**0.004**
Duration of third stage (minute)	17.8 ± 7.5	17.2 ± 7.8	0.118
Hb of before labour (g/dl)	10.5 ± 0.8	10.7 ± 0.9	0.318
Hb of after labour (g/dl)	9.5 ± 0.9	10.1 ± 1.0	**0.042**
APGAR at 1st minute	5.9 ± 1.6	6.9 ± 1.1	**0.031**
APGAR at 5th minute	8.3 ± 2.0	8.7 ± 1.9	**0.048**
Birth weight (gram)	3173.1 ± 477.2	3199.7 ± 508.3	0.318

Bold values indicate* p* < 0.05

BMI: Body mass index, Hb: Hemoglobin

*Student T-Test

There was no difference between groups in terms of primigravida status. Stillbirth, outlet obstruction, perineal tear, need for cesarean section, need for neonatal intensive care unit, and newborn APGAR score below 7 were found to be significantly higher in the FGM group. We showed that obstetric and neonatal outcomes at birth and afterwards in Table 3.

**Table 3:** Obstetrics and neonatal outcomes

	FGM group (*n* = 134) Number (%)	Control group (*n* = 134) Number (%)	RR (95% Cl)	*p* value*
Stillbirth	4 (2.98)	2 (1.49)	n.a**	**0.038**
Outlet obstruction	42 (31.34)	18 (13.43)	2.33 (1.21–3.45)	**0.022**
Perineal tear	21 (15.67)	9 (6.71)	2.48 (1.28–3.68)	**0.033**
Need for cesarean section	19 (14.17)	9 (6.71)	2.11 (1.05–3.17)	**0.036**
APGAR score < 7 at 5th minute	12 (8.95)	6 (4.47)	2 (0.98–3.02)	**0.042**
Need for neonatal intensive care unit of newborns	15 (11.19)	8 (5.97)	1.87 (0.89–2.85)	**0.047**
Primigravida	54 (40.29)	52 (38.80)	n.a	0.348

Bold values indicate* p* < 0.05

*Chi-square test

**RR was not specified due to the small number

## Discussion

In our study, we aimed to investigate whether female genital mutilation affects birth complications and neonatal outcomes in Somalia. In this study, although there was no significant difference in other stages of labour in women with FGM, there was a significant prolongation in the second stage of labour. Gayle et al. found that prolongation of the duration of labour and an increase in the frequency of caesarean section women with FGM [17].

There are many studies in which postpartum hemorrhage is more common in women with FGM [7, 17, 18]. In our study, although Hb levels were similar between the prenatal groups, we found significantly lower levels in the postnatal FGM group.

The weight of the baby at birth affects birth complications [19, 20]. In our study, no difference was found between the two groups in terms of infant birth weight.

In the literature, there are many studies showing that the need for emergency cesarean section during delivery is high in patients with FGM [9, 12]. In our study, we found that women with FGM needed more emergency cesarean section during labour.

There are studies showing that the risk of stillbirth increases with FGM [7, 12]. We found that the risk of stillbirth increased in the group with FGM, which was consistent with the literature. In the study of Gebremicheal et al., it was determined that there was a 2.38-fold increase in the risk of outlet obstruction in patients with FGM. In our study, we found a 2.33-fold increase in this risk in patients with FGM.

Scarred perineal tissue, increased fibrosis, and decreased elasticity were observed with FGM, resulting in an increased incidence of episiotomy [21]. It has been reported that the risk of perineal tear increases by at least 50% because of the loss of elasticity of the perineum with FGM [9, 12, 21]. In our study, we found statistically significantly more perineal tears in women with FGM in line with the literature.

Kaplan et al. reported that the need for NICU increased in women with FGM [21]. We found that the risk of needing NICU in newborns in the FGM group was significantly increased compared to the control group. FGM, which has negative effects on newborn well-being [22], also significantly affected APGAR scores in the current study. We found both 1st and 5th minute APGAR scores to be lower in the group with FGM. In our study, the risk of having APGAR score < 7 at 5th minute increased 2 times compared to the control group.

WHO defines the stillbirth is defined as fetal death occurring after 28 weeks of gestation [23]. While there is 1 stillbirth in 160 births in the United States, it is estimated that there are 2.6 million stillbirths in the world each year [23, 24]. Many causes of stillbirth appear to be preventable[23]. FGM has been shown in some studies as one of those reasons. Although stillbirth was seen to be higher in women with FGM, it was not detected as a risk factor in the study of Andro et al. [25–27]. The inability to calculate the stillbirth risk, which is very rare in our study, was a limitation. However, we conducted this study with a reasonable number of participants in a place where the health registration system is not developed enough, such as in Somalia.

Some studies in the literature associated obstetric complications more with type 2 or type 3 FGM [9, 28]. In our study, the majority (61%) of the FGM group comprised patients with type 2 FGM, 19% of them were patients with type 3 FGM, and 20% of patients with type 1 FGM. Although calculating the frequency of complications according to FGM types was considered a limitation, most of the pregnant women in our study consisted of women with type 2 and type 3 FGM.

## Conclusions

This study showed that FGM leads to prolongation of the second stage of labour, increased risk of perineal tear, increased need for emergency caesarean section, and increased NICU need in infants. FGM is still widely practiced in countries such as Somalia. It would be beneficial to take steps with both international and local authorities to completely abolish FGM. With its negative consequences, FGM not only affects the health of women and children, but also creates an unnecessary economic burden for the states.

## Data Availability

The datasets generated and/or analyzed during the current study are not publicly available because of our hospital policy, but are available from the corresponding author on reasonable request.
